# Real flue gas CO_2_ hydrogenation to formate by an enzymatic reactor using O_2_- and CO-tolerant hydrogenase and formate dehydrogenase

**DOI:** 10.3389/fbioe.2023.1265272

**Published:** 2023-10-03

**Authors:** Jaehyun Cha, Jinhee Lee, Byoung Wook Jeon, Yong Hwan Kim, Inchan Kwon

**Affiliations:** ^1^ School of Materials Science and Engineering, Gwangju Institute of Science and Technology (GIST), Gwangju, Republic of Korea; ^2^ School of Energy and Chemical Engineering, Ulsan National Institute of Science and Technology (UNIST), Ulsan, Republic of Korea; ^3^ Research Center for Innovative Energy and Carbon Optimized Synthesis for Chemicals (Inn-ECOSysChem), Gwangju Institute of Science and Technology (GIST), Gwangju, Republic of Korea

**Keywords:** carbon dioxide, hydrogen, formate, flue gas, hydrogenase, formate dehydrogenase

## Abstract

It is challenging to capture carbon dioxide (CO_2_), a major greenhouse gas in the atmosphere, due to its high chemical stability. One potential practical solution to eliminate CO_2_ is to convert CO_2_ into formate using hydrogen (H_2_) (CO_2_ hydrogenation), which can be accomplished with inexpensive hydrogen from sustainable sources. While industrial flue gas could provide an adequate source of hydrogen, a suitable catalyst is needed that can tolerate other gas components, such as carbon monoxide (CO) and oxygen (O_2_), potential inhibitors. Our proposed CO_2_ hydrogenation system uses the hydrogenase derived from *Ralstonia eutropha* H16 (ReSH) and formate dehydrogenase derived from *Methylobacterium extorquens* AM1 (MeFDH1). Both enzymes are tolerant to CO and O_2_, which are typical inhibitors of metalloenzymes found in flue gas. We have successfully demonstrated that combining ReSH- and MeFDH1-immobilized resins can convert H_2_ and CO_2_ in real flue gas to formate via a nicotinamide adenine dinucleotide-dependent cascade reaction. We anticipated that this enzyme system would enable the utilization of diverse H_2_ and CO_2_ sources, including waste gases, biomass, and gasified plastics.

## 1 Introduction

Global warming is an issue that has garnered significant attention in recent years due to its potentially catastrophic impacts on the environment and human societies (Frölicher et al., 2018; Hughes et al., 2018). One of the primary causes of global warming is the release of carbon dioxide (CO_2_) into the atmosphere, mainly due to human activities such as burning fossil fuels (Salam and Noguchi, 2005; Armaroli and Balzani, 2011; Olah et al., 2011; Al-Ghussain, 2019). To mitigate the effects of global warming, it is crucial to develop effective strategies for reducing CO_2_ emissions (Takemura and Suzuki, 2019). Due to its stability as a fully oxidized gaseous molecule at room temperature and pressure, capturing CO_2_ from the atmosphere presents a formidable challenge (Rochelle, 2009; Ghalei et al., 2017; Ding and Jiang, 2018). Moreover, as large-scale captured CO_2_ holds little industrial value, the most promising approach to regenerate combusted CO_2_ is through CO_2_ hydrogenation (Li et al., 2018; Ye et al., 2019).

One potential approach to CO_2_ hydrogenation is the conversion of CO_2_ and hydrogen (H_2_) into formate, using hydrogen as a renewable energy source (Wang et al., 2015; Wang et al., 2019). Formate, one of the most basic C1 compounds, exists as a non-flammable liquid at ambient temperature and pressure, serving as both a precursor for various syntheses and a primary storage medium for captured CO_2_ (Kothandaraman et al., 2015). Further hydrogenation of formate can lead to the production of formaldehyde or methanol (Wang et al., 2015), while microorganisms can utilize it to generate biopolymers (Hwang et al., 2020). However, to enable the hydrogenation of a massive amount of CO_2_, cheap H_2_ derived from sustainable sources is required.

Industrial flue gas can address this issue as a cheap H_2_ source. According to an International Energy Agency report, around 30% of industrial CO_2_ emissions came from the steel industry (Kim et al., 2019). The steel mill generates various flue gases such as coke oven gas (COG), blast furnace gas (BFG), and Lintz-Donawitz gas (LDG) (Lee et al., 2020). In particular, COG has a high H_2_ content of nearly 60% (Moral et al., 2022) (Table 1). These gases are promising cheap H_2_ resources but are used inefficiently for auxiliary facilities in steel mills as heat or power sources for boilers (Xiang et al., 2016). While it is unsurprising that efforts have been made to harness this H_2_ for CO_2_ hydrogenation, the presence of multiple gases in the industrial flue gas necessitates the use of a catalyst that is practically free of side reactions and tolerant to other gas components like carbon monoxide (CO) and oxygen (O_2_). To address this issue, we propose a CO_2_ hydrogenation system that utilizes multiple enzymes, taking advantage of their high reactivity and substrate specificity under ambient temperature and pressure conditions.

**TABLE 1:** Composition of flue gases from Hyundai Steel^a^.

		Coke oven gas (COG)	Blast furnace gas (BFG)	COG + BFG (1:1 mix)	Lintz-donawitz gas (LDG)
Component (v/v, %)	H_2_	55.4 ± 0.5	3.8 ± 0.2	29.6 ± 0.5	1.4 ± 0.4
	CO_2_	2.4 ± 0.1	23.6 ± 0.2	13 ± 0.2	19.3 ± 0.6
	CO	6.2 ± 0.1	25.5 ± 0.3	15.9 ± 0.3	49.4 ± 1.9
	O_2_	1.1 ± 0.02	1.1 ± 0.00	0.6 ± 0.02	0.01 ± 0.02
	N_2_	8.6 ± 0.6	46.7 ± 0.1	27.7 ± 0.6	29.2 ± 1.9
	CH_4_	24.2 ± 0.4	-	12.1 ± 0.4	-
	C_m_H_n_	3.1 ± 0.1	-	1.6 ± 0.1	-

^a^ Data of Hyundai Steel are calculated from the previous studies (Kim et al., 2022).

The CO_2_ hydrogenation reaction occurs as two half-reactions: H_2_ oxidation and CO_2_ reduction (equation (1)-3) (Loges et al., 2008; Reda et al., 2008). The enzymes responsible for each reaction are hydrogenase (H_2_ase) for H_2_ oxidation (Lubitz et al., 2014) and formate dehydrogenase (FDH) for CO_2_ reduction (Appel et al., 2013; Amao, 2018; Moon et al., 2020). The conversion of H_2_ and CO_2_ to formate was reported through a cascade reaction involving the reaction of H_2_ase and FDH and electron transfer (Sokol et al., 2019). Flue gas contains CO, O_2_, nitrogen (N_2_), *etc.*, in addition to the substrate H_2_ and CO_2_. The H_2_ase and FDH used in the previous study as well as most other H_2_ases and FDHs were derived from anaerobic microorganisms, and so their metal-active sites are often attacked and inactivated even in the presence of trace amounts of O_2_ (Fontecilla-Camps et al., 2007; Niks and Hille, 2018). CO also acts as an irreversible inhibitor of most H_2_ases (Bagley et al., 1994; Vincent et al., 2007).

$$2H^{+}\;⇆\;H_{2}\;(E{˚}^{\prime }\left.=-0.382\;V\text{ vs}.\text{ SHE},\text{pH }6.5\right)$$ (1)

$${\text{CO}}_{2}+H^{+}\;⇆\;{\text{HCO}}_{2}^{-}\left(E\right.{˚}^{\prime }\left.=-0.366\;V\text{ vs}.\text{ SHE},\text{pH }6.5\right)$$ (2)

$${\text{CO}}_{2}+H_{2}\;⇆\;{\text{HCO}}_{2}^{-}+H^{+}\;(E˚\prime_{\text{rxn}}=U˚\prime \left.=0.016\;V\right)$$ (3)

We previously demonstrated the feasibility of the combined use of O_2_-tolerant H_2_ase and FDH using pure gases (Cha et al., 2023a). However, for CO_2_ hydrogenation using the real flue gas, several issues should be solved. First, with the ultimate goal of long-term repeated or continuous reactions, enzymes should be immobilized on solid supports. We developed the suitable pair of immobilization tag and affinity resin for each enzyme. Second, the enzymes need to be tolerant to both CO and O_2_. Therefore, we investigated the impact of CO or O_2_ on the kinetic parameters of both H_2_ase and FDH. Third, the H_2_ase and FDH, capable of electron transfer between the two reactions, must satisfy catalytic bias. Therefore, we selected H_2_nase and FDH, which exhibit a catalytic bias towards a desired direction to the greatest extent possible.

First, we chose the hydrogenase from *Ralstonia eutropha* H16 (ReSH) consisting of heterodimeric [NiFe] hydrogenase (HoxHY) subunits and diaphorase (HoxFU) subunits, stably oxidized H_2_ and reduced NAD^+^ to NADH under oxic conditions (Lauterbach and Lenz, 2013; Wulff et al., 2014; Horch et al., 2015). In addition, it has also been reported that ReSH is not sensitive to CO, unlike other hydrogenases (Burgdorf et al., 2005). Resistant to O_2_ and CO, ReSH is a promising H_2_ase candidate that can reduce NAD^+^ to NADH by consuming hydrogen in real flue gas. FDHs are classified as metal-independent or metal-dependent based on the active site (molybdenum or tungsten) (Ünlü et al., 2021; Alpdağtaş et al., 2022). Although metal-independent FDH exhibits O_2_-independent activity, the strategies like mutation and immobilization have been reported to enhance its activity and stability due to its inherently low activity (Çakar et al., 2020; Tülek et al., 2023). FDHs from *Clostridium carb*oxidovorans strain P7T (Alissandratos et al., 2013), *Chaetomium thermophilum* (Çakar et al., 2020), *Rhodobacter capsulatus* (Hartmann and Leimkühler, 2013), and *Methylobacterium extorquens* AM1 (Laukel et al., 2003) have maintained high CO_2_ reduction activity under oxic conditions. Second, we chose tungsten-containing FDH1 from *M. extorquens* AM1 (MeFDH1), consisting of alpha and beta subunits, as FDH to produce formate via enzymatic NAD^+^ regeneration stably (Baccour et al., 2020). It was reported that MeFDH1 has the great catalytic bias towards CO_2_ reduction. The resistance of MeFDH1 to CO has not been reported.

**FIGURE 1:**
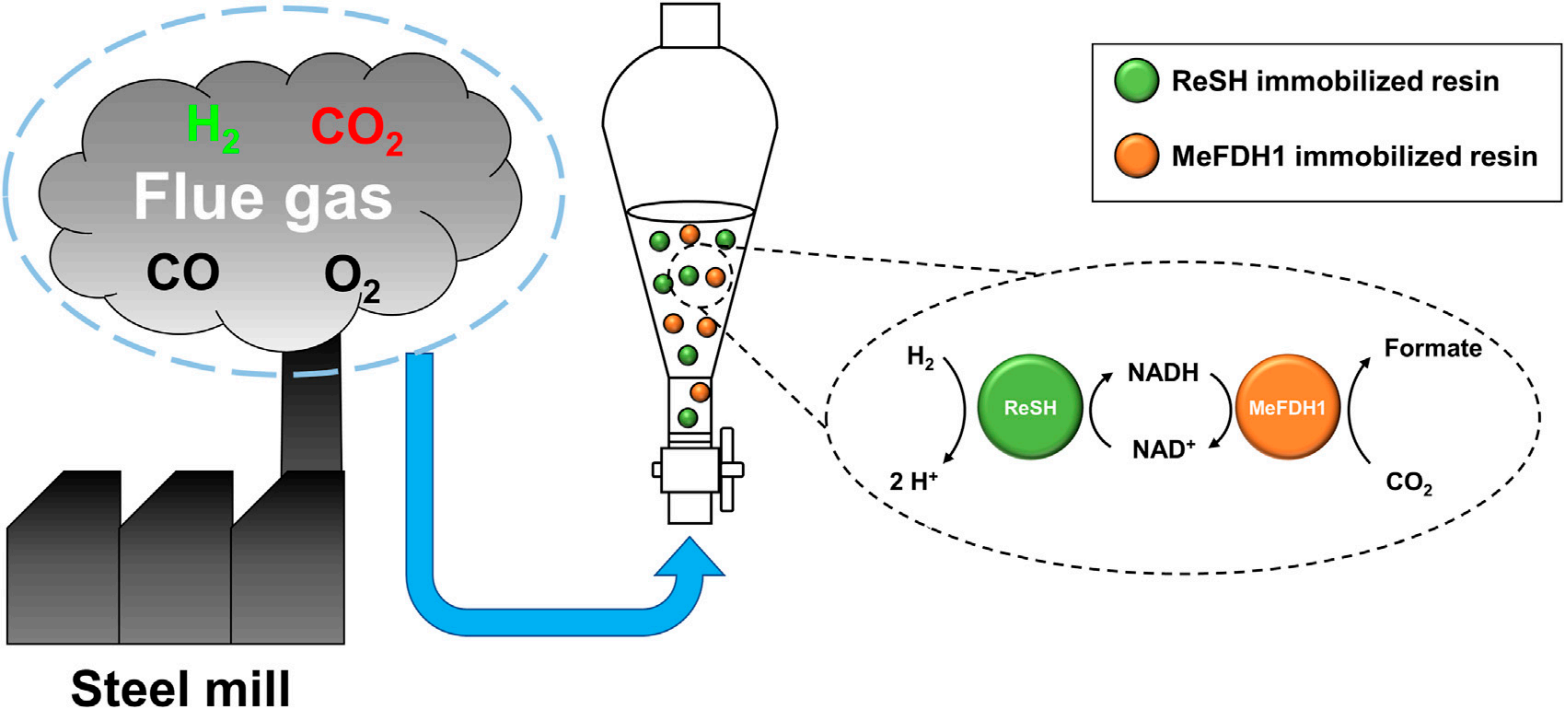
Schematic showing an enzymatic reactor for flue gas conversion. The reactor converts H_2_ and CO_2_ to formate via an NAD^+^-dependent cascade reaction of immobilized ReSH and MeFDH1.

In this study, we constructed an enzymatic reactor with ReSH-immobilized resins, MeFDH1-immobilized resins, and NAD^+^, which showed CO_2_ hydrogenation from real flue gas (Figure 1). The kinetic parameters were determined to ensure each enzyme could withstand the potential critical inhibitors O_2_ and CO in the flue gas environment. The gaseous substrate needs to be bubbled to supply the enzymatic reactor continuously. To reduce aeration damage, the reactor was constructed with the enzymes immobilized on an agarose based affinity resin, respectively.

## 2 Materials and methods

### 2.1 Materials

COG and BFG were supplied by Hyundai steel (Ulsan, South Korea). Ni-NTA agarose and a gravity-flow polypropylene column (PP column, 10 mL) were purchased from Qiagen (Hilden, Germany). Strep-Tactin XT 4 Flow high-capacity resin was obtained from IBA Life Sciences (Göttingen, Germany). Disposable PD-10 desalting columns were purchased from Cytiva (Marlborough, MA, United States). Unless otherwise stated, all other chemical reagents were purchased from Sigma-Aldrich (St. Louis, MO, United States).

### 2.2 Preparation of immobilized and purified ReSH and MeFDH1

Strains and expression conditions of ReSH and MeFDH1 were described in previous studies (Jang et al., 2018; Cha et al., 2023a). To purify ReSH, cell pellets were resuspended in 50 mM potassium phosphate (Kpi) buffer (pH 7.0) containing 1 mg/mL lysozyme to a concentration of 1 g/10 mL. The resuspended cells were lysed by sonication (amplitude 28%, on/off 2 s/4 s) for 1 h. Insoluble cell debris was removed by centrifugation at 13,000 × g for 30 min. Strep-Tactin XT 4Flow high-capacity resin was mixed with the clear supernatants incubated at 4 °C for 30 min. The resin was washed with 50 mM Kpi buffer (pH 7.0) containing 300 mM potassium chloride on a PP column to remove impurities. After running off the wash solution, plug the end of the pp column, add 200 mM Kpi (pH 6.5), and resuspended the resin to obtain the ReSH immobilized resin for cascade reactions. The ReSH-immobilized resin was stored at 4°C until use. In order to obtain purified ReSH, the resin was eluted with 3 mL of 200 mM Kpi (pH 6.5) containing 50 mM biotin and buffer-exchanged with 200 mM Kpi buffer (pH 6.5) using a PD-10 column.

The purification of MeFDH1 was started with the cell lysis by sonication in an anaerobic chamber. The wet cell pellet was suspended into the buffer A (50 mM (3-(N-morpholino) propanesulfonic acid (MOPS), 200 mM NaCl, 20 mM imidazole, 2 mM dithioerythritol (DTE) and 2 µM resazurin, pH 7.0) with 1 g wet cell/10 mL buffer concentration and sonicated (amplitude 35%, on/off 2 s/2 s). The lysate was centrifuged at 11,000 rpm at 4 °C for 20 min. The supernatant and Ni-NTA agarose bead were mixed together and incubated for 15 min to bind protein. The protein-bound Ni-NTA agarose bead was separated from the mixture using a gravity flow column. The collected protein-bound Ni-NTA agarose bead was washed with buffer A. The MeFDH1-bound Ni-NTA agarose bead was stored at 4°C until use. For the activity and kinetic property assay, the purified MeFDH1 was eluted by buffer B (50 mM MOPS, 200 mM NaCl, 300 mM imidazole, 2 mM DTE and 2 µM resazurin, pH 7.0).

Protein purity was verified by SDS-PAGE (Figs. Sa and b). The concentrations of purified ReSH was determined by measuring their absorbance at 280 nm using a microplate reader (Synergy, BioTek, Winooski, VT, United States), as previously reported for other proteins (Bak et al., 2020; Kim et al., 2021). The concentrations of purified MeFDH1 was determined by measuring their absorbance at 280 nm using a NanoDrop 1 C (Thermo fisher scientific, Waltham, Massachusetts, United States). The extinction coefficients of ReSH and MeFDH1 were calculated to be 165,710 and 153,735 M^−1^⋅cm^−1^, respectively, based on their amino acid sequences.

### 2.3 Enzyme kinetics

The enzyme reaction kinetic parameters of ReSH were measured for the NAD^+^-dependent oxidation of H_2_ to H^+^ in the presence or absence of O_2_ and CO. The sealing cuvette was filled with 900 μL of 200 mM Kpi buffer (pH 6.5) containing NAD^+^ and sealed; then, 100% H_2_ and mix gas containing 1) 100% N_2_, 2) 96% N_2_ and 4% O_2_ 3) 60% N_2_ and 40% CO, and 4) 56% N_2_, 4% O_2_ and 40% CO were injected simultaneously for 30 min at 10 mL/min. ReSH (6 mL, 80 nM) was purged with 10 mL/min N_2_ gas bubbling in a 25 mL sealing vial for 30 min to remove O_2_ from the air. The reaction was initiated by mixing 100 μL of 80 nM ReSH with a gas-saturated solution in a sealed cuvette. The final concentration of NAD^+^ was varied from 0 to 4 mM.

The enzyme reaction kinetic parameters of MeFDH1 were measured for the NADH-dependent reduction of CO_2_ to formate in the presence or absence of O_2_ and CO. The 200 mM Kpi buffer (pH 6.5) was purged with gases containing 1) 100% N_2_, 2) 100% O_2_ and 3) 100% CO for 1 h in closed serum bottles. Each gas-purged buffer was mixed to make 1) 100% N_2_, 2) 98% N_2_ and 2% O_2_, 3) 80% N_2_ and 20% CO and 4) 78% N_2_, 20% CO and 2% O_2_ dissolved buffers. The powder of sodium bicarbonate and NADH was dissolved into each buffers (100 mM of sodium bicarbonate and 6.25—100 µM of NADH). The 980 μL of buffers are finally aliquoted into the cuvettes and sealed. By injecting 10 ug of MeFDH1 in 20 uL of buffer B, the reaction was started.

All measurements were performed in triplicate based on the change in the absorbance at 340 nm in the cuvette. The absorbane changes for ReSH and MeFDHS1 were measured using the T60 UV-Vis spectrophotometer (PG Instruments Ltd., Lutterworth, UK)and the UV-1650 PC (Shimadzu, Kyoto, Japan), respectively. The inverse of initial reaction rates was plotted *versus* the substrate concentration according to the Hanes-Woolf plot to calculate the kinetic parameters.

### 2.4 Formate production and quantification

For the cascade reaction, the gas content was controlled in a gas flow reactor (Figure 1). The reactor was filled with 6 mL of reactor containing 20 U of immobilized ReSH, 1 U of immobilized MeFDH1, 1 mM NAD^+^, and 1% (v/v) antifoam A. All components were resuspended or dissolved in 200 mM Kpi buffer (pH 6.5). The reaction was initiated by a 30 mL/min substrate gas injection. Formate production was sampled every 30 min during incubation for 1.5 h, and 20 μL of 6 N H_2_SO_4_ was added to the 200 μL sample to inactivate the enzymes immediately. Additionally, 180 μL of distilled water was mixed with the sample, and the aggregated enzymes were removed by syringe filter. Formate production was quantified by HPLC (1,260, Agilent, CA, United States) equipped with a diode-array detector and an Aminex HPX-87H column (BIO-RAD, CA, United States) with a mobile phase of 5 μM H_2_SO_4_ at a flow rate of 0.6 mL/min.

## 3 Results and discussion

### 3.1 Preparation of ReSH and MeFDH1

Recombinant ReSH and MeFDH1 were expressed in *R. eutropha* and *M. extorquens*, respectively. The ReSH and MeFDH1 were immobilized on the strep tactin XT 4flow resin and Ni-NTA agarose affinity resins, respectively, as described in the Materials and Methods section. A fraction of these was eluted separately to obtain purified proteins. The SDS-PAGE analysis was performed to verify the existence of subunits and purity of each. Five bands of purified ReSH subunits were observed, which matched their expected molecular weights (HoxF, 68,110 Da; HoxH, 54,863 Da; HoxU, 26,173 Da; HoxY, 22,881 Da; HoxI, 18,567 Da) (Supplementary Figure S1a). Two bands of purified MeFDH1 subunits were also observed, consistent with their expected molecular weights (FdhA1: 107,341 Da, FdhA2: 62,321 Da) (Supplementary Figure S1b). The purity of both enzymes was high. To calculate the enzyme units of ReSH- or MeFDH1 immobilized resin, we measured the concentration and enzymatic activity of purified ReSH and MeFDH1, respectively. The ReSH-immobilized resin had an NAD^+^-dependent H_2_ oxidation activity of 140.9 U/mL. The MeFDH1-immobilized resin had an NADH-dependent CO_2_ reduction activity of 0.53 U/mL. These results showed that the immobilized ReSH and MeFDH1 were successfully prepared.

### 3.2 pH-dependency of enzymes

**FIGURE 2:**
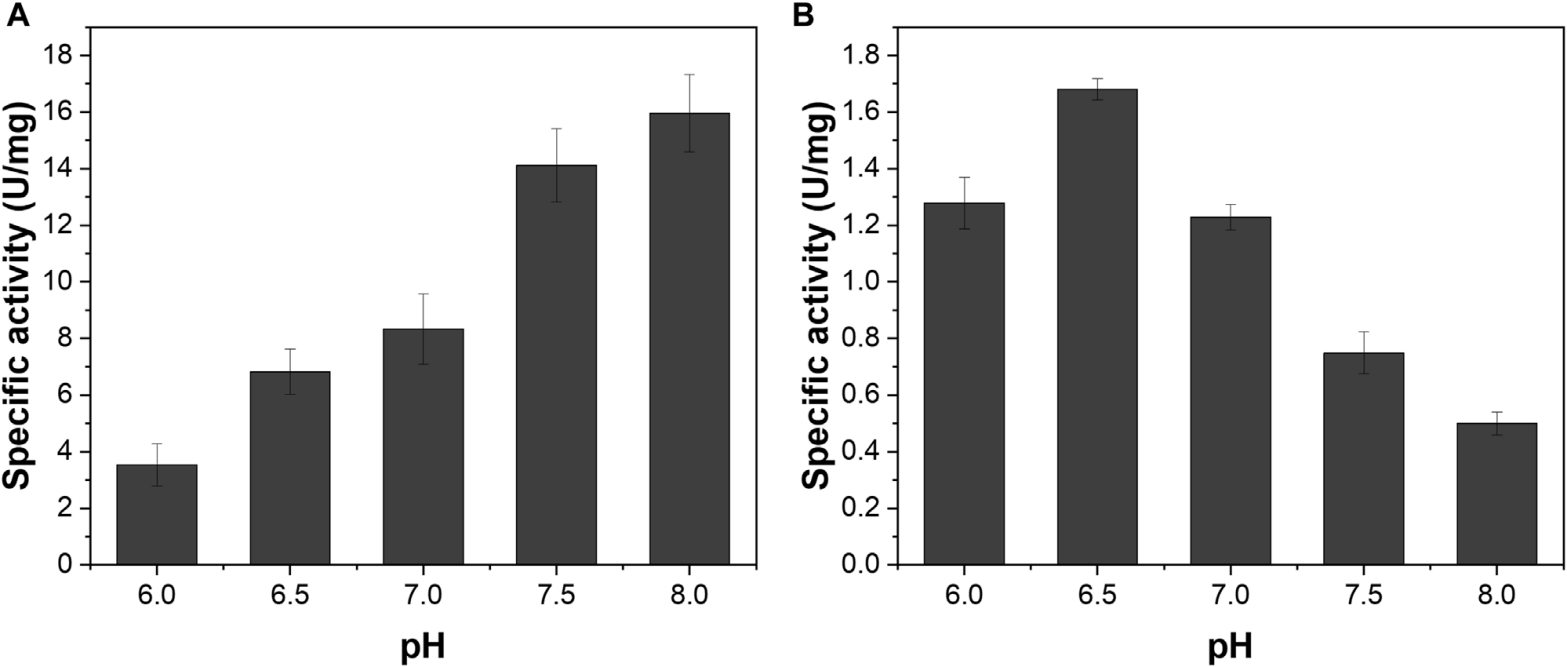
pH-dependency of **(A)** ReSH and **(B)** MeFDH1 activity. The enzyme activities were determined in the pH range of 6.0–8.0 in 200 mM Kpi buffer.

We investigated the pH-dependent enzyme activities of NAD^+^-dependent H_2_ oxidation of ReSH and NADH-dependent CO_2_ reduction of MeFDH1 to determine the buffer conditions in the reactor for the cascade reaction. The specific activity of ReSH tended to increase with increasing pH from 6 to 8 (Figure 2A). In contrast, the specific activity of MeFDH1 tended to decrease with increasing pH, with the maximum activity at pH 6.5 (Figure 2B). Since the specific activity of the two enzymes is maximized at different points, we decided to use pH 6.5 as the cascade reaction condition where the activity of MeFDH1, which produces formate, is maximal.

### 3.3 Enzyme kinetics in the flue gas mimic conditions

We investigated whether both ReSH and MeFDH1 can tolerate the potential inhibitors (O_2_ and CO) present in the flue gas at pH 6.5. The final concentrations of O_2_ and CO were 2% and 20%, respectively, which is higher than those inside the COG, to evaluate enzyme activity inhibition. The NAD^+^-dependent H_2_ oxidation reaction rate by ReSH was measured, and the Hanes-Woolf plot was fitted to calculate the kinetic parameters using Origin 2022 program (Supplementary Figure S2). The *k*_*cat*_ and *K*_*m*_ values of ReSH were not substantially different in four conditions: control without any O_2_ and CO, 2% O_2_, 20% CO, and both 2% O_2_ and 20% CO (Table 2). Similarly, The NADH-dependent CO_2_ reduction reaction rate by MeFDH1 was measured, and the Hanes-Woolf plot was fitted to calculate the kinetic parameters (Supplementary Figure S3). The *k*_*cat*_ and *K*_*m*_ of MeFDH1 were not substantially different in four conditions: control without any O_2_ and CO, 2% O_2_, 20% CO, and both 2% O_2_ and 20% CO (Table 3). These results show that at concentrations below 2% O_2_ and 20% CO, the enzymatic activities of ReSH and MeFDH1 are not substantially inhibited, indicating their potential for use in flue gas conversion.

**TABLE 2:** Kinetic parameters of ReSH in the presence of O_2_ or CO.

Gas contents	*k*_*cat*_ (s^-1^)	*K*_*m*_ (μM) (NAD^+^)	*k*_*cat*_/*K*_*m*_ (μM^-1^ s^-1^)
50% H_2_, 50% N_2_	55.22 ± 0.82	1,179.4 ± 47.1	0.0468 ± 0.0020
50% H_2_, 2% O_2_	54.25 ± 1.29	1,162.4 ± 75.4	0.0467 ± 0.0032
50% H_2_, 20% CO	54.08 ± 3.68	1,146.3 ± 205.2	0.0472 ± 0.0090
50% H_2_, 2% O_2_, 20% CO	57.11 ± 3.49	1,249.9 ± 149.7	0.0457 ± 0.0061

**TABLE 3:** Kinetic parameters of MeFDH1 in the presence of O_2_ or CO.

Gas contents	*k*_*cat*_ (s^-1^)	*K*_*m*_ (μM) (NAD^+^)	*k*_*cat*_/*K*_*m*_ (μM^-1^ s^-1^)
100% Carbonate	2.272 ± 0.064	26.38 ± 1.16	0.0862 ± 0.0030
2% O_2_	2.232 ± 0.029	26.15 ± 1.02	0.0854 ± 0.0023
20% CO	2.237 ± 0.052	24.79 ± 1.12	0.0903 ± 0.0026
2% O_2_, 20% CO	2.276 ± 0.064	25.97 ± 1.76	0.0878 ± 0.0038

### 3.4 Enzymatic reactor optimization

**FIGURE 3:**
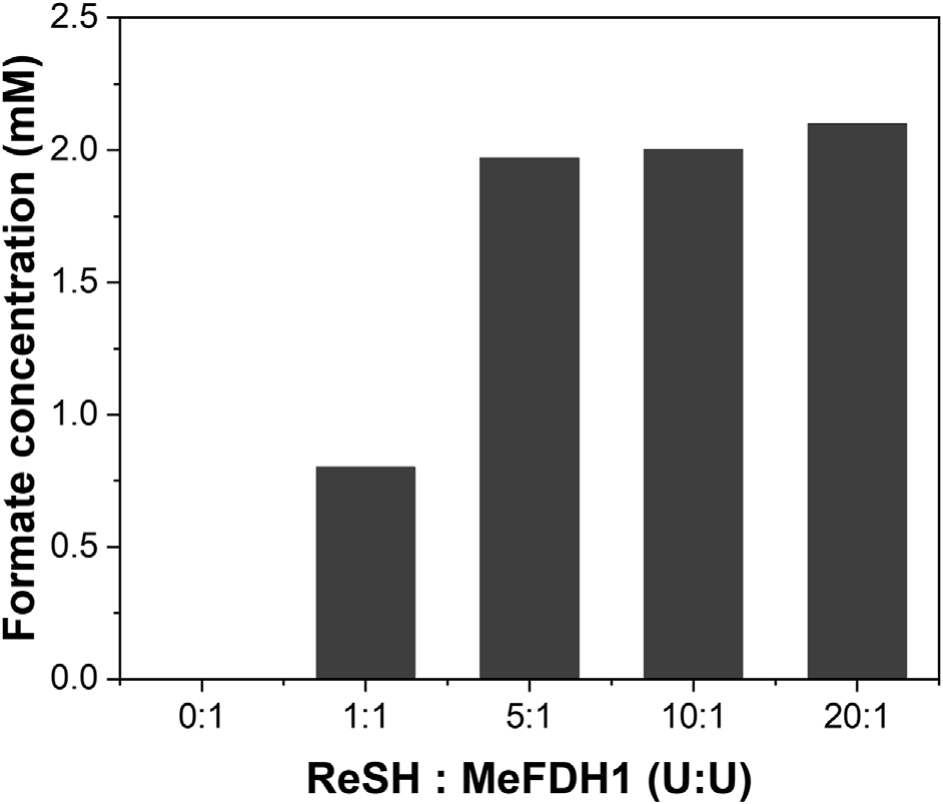
Formate produced during 1 h at different ratios of ReSH to MeFDH1. The activity of ReSH was varied with 0, 1, 5, 10, and 20 U (ReSH:MeFDH1 = 0:1, 1:1, 5:1, 10:1, 20:1) in the presence of 1 U MeFDH1 and 1 mM NAD^+^.

We optimized the components of the enzymatic reactor for a smooth cascade reaction. The reaction mechanism is that ReSH oxidizes H_2_, and NAD^+^ is reduced to NADH, which is then oxidized back to NAD^+^ by MeFDH1, which reduces CO_2_ to formate. Since MeFDH1 has a *K*_*m*_ value of about 25 uM, we used 1 mM of NAD^+^ as a sufficient initial input to produce the formate at V_max_. For formate production to continue at the maximum rate with the minimal reverse reaction of MeFDH1, NAD^+^ must be reduced immediately so that NADH is dominant during the reaction. To achieve this, the enzyme unit of ReSH must be higher than that of MeFDH1. We fixed the MeFDH1 immobilized resin at 1 U and varied the ReSH immobilized resin from 0 to 20 U (Unit ratio ReSH: MeFDH1 = 0:1 to 20:1) and measured the formate produced when supplied with gas substrate H_2_ and CO_2_ for 1 h (Figure 3). Formate production was quantified via HPLC. The retention time of formate was 13.010 min (Cha et al., 2023a). No formate was produced in the reactor without ReSH. Compared to the 1:1 ReSH to MeFDH1 unit ratio, all experiments with an excess of ReSH produced about 2 mM of formate, and experiments with an excess of ReSH over 5:1 tended to saturate formate production. Based on these results, we determined that the unit ratio of ReSH to MeFDH1 was 20:1 for sufficient formate productivity.

### 3.5 Formate production from flue gas mimic condition

**FIGURE 4:**
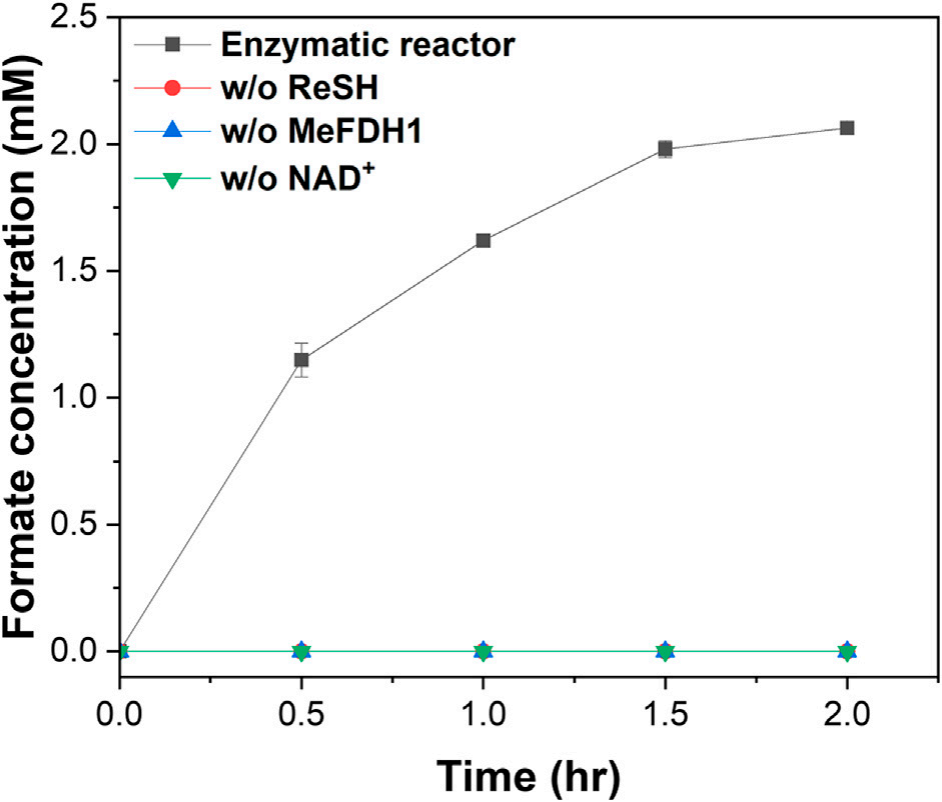
Formate production by enzymatic reactors in the presence of O_2_ and CO. As negative controls, enzymatic reactors were constructed lacking ReSH, MeFDH, and NAD^+^, respectively. The flue gas mimic containing 30% H_2_, 15% CO_2_, 15% CO, 2% O_2_, and 38% N_2_ were fed to the enzymatic reactor at 30 mL/min for 2 h. All measurements were performed in triplicate.

We demonstrated CO_2_ hydration using a flue gas mimic. We decided to implement a specific flue gas mimic condition by mixing different syngas, but most flue gases have a high percentage of either H_2_ or CO _2_. For example, COG had more than 50% H_2_ but less than 3% CO_2_, and BFG had 20% CO_2_ but less than 4% H_2_. So the flue gas mimic realized them in a ratio similar to the gas contents after mixing them 1:1 (Table 1). 20 U of the ReSH immobilized resin and 1 U of the MeFDH1 immobilized resin and 1 mM NAD^+^ were mixed and placed in a gas flow reactor. Formate production was monitored for 2 h at 30 min intervals with a continuous flow of flue gas mimic to the enzymatic reactor containing a mixture of all reaction components (Figure 4). No formate was detected in the negative control group, missing one of ReSH, MeFDH1, and NAD^+^. Following the trend of previous kinetics, we found that formate was produced in 2% O_2_ and 20% CO. The formate tended to reach about 2 mM around 2 h and then saturate without further increase. We speculated that the elevated formate concentration increased the reverse reaction rate, causing it to reach equilibrium.

### 3.6 Formate production using real flue gases

**FIGURE 5:**
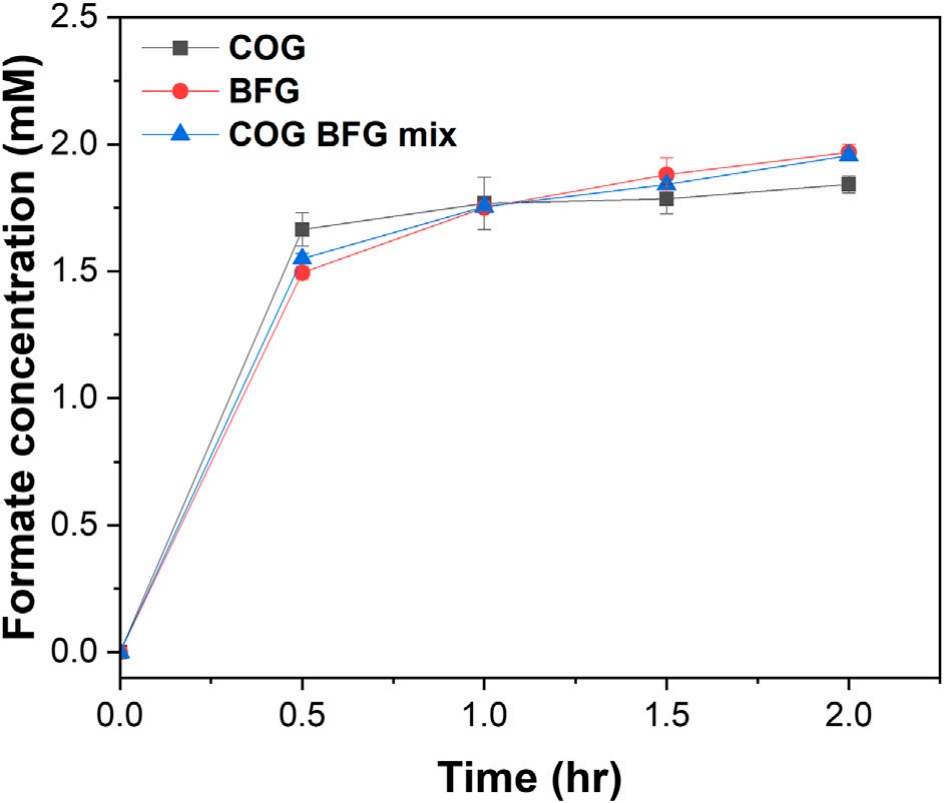
Formate production by the enzymatic reactors using real flue gas. COG, BFG, and 1:1 mixture of COG and BFG were fed to the enzymatic reactor at 30 mL/min for 2 h. All measurements were performed in triplicate.

We investigated formate production by feeding real flue gases to the enzymatic reactor. We provided the COG, BFG, and a 1:1 mix of COG and BFG to the enzymatic reactor and monitored the formate production (Figure 5). Contrary to our expectations, both COG, which is rich in H_2_ and relatively low in CO_2_, and BFG, which is low in H_2_ and high in CO_2_, saturated in formate production around 2 mM at 2 h, just like the flue gas mimic, and we saw the same trend in formate production when we fed a 1:1 mixture of the two gases. We speculated that even at relatively low concentrations of H_2_ in BFG or CO_2_ in COG, the reaction rates of ReSH and MeFDH1 were high enough to have a V_max_ due to the continuous gas flow. In practice, however, CO_2_ and H_2_ react in a 1:1 mole ratio, so mixing the flue gases to produce formate, as we have shown, is a significant step toward industrial-scale CO_2_ hydration. However, to solve the current situation where formate production is saturated, it is necessary to configure a repeated batch or continuous flow reactor to separate the formate produced from the reactor continuously. As described in the Introduction section, FDHs for enzymatic CO_2_ reduction are categorized into metal-independent and metal-dependent types. Metal-independent FDHs are not affected by O_2_ but generally exhibit relatively low catalytic activities (with a range of *k*_*cat*_/*K*_*m*_ 0.0004–0.08 s^-1^ mM^-1^) (Alpdağtaş et al., 2022). On the other hand, metal-dependent FDHs display high catalytic activity (with a range of *k*_*cat*_/*K*_*m*_ 10^0^∼10^3^ s^-1^ mM^-1^), but most of them face difficulties in utilizing real CO_2_ sources due to susceptibility to inactivation by O_2_ (Moon et al., 2020; Calzadiaz-Ramirez and Meyer, 2022). The MeFDH1 enzyme used in this study is expected to serve as a compromise, as it demonstrates catalytic activity falling between these two ranges and displays resistance to both O_2_ and CO.

## 4 Conclusion

We have successfully demonstrated CO_2_ hydrogenation using hydrogen in the real flue gas from the steel industry via an NAD^+^-dependent cascade reaction in an enzymatic reactor that combines a specific H_2_ase and FDH. We confirmed that ReSH and MeFDH1, previously reported as O_2_-tolerant, are also resistant to CO, a potential inhibitor of metalloenzymes. We used both H_2_ and CO_2_ from the flue gas in this study. However, the enzymatic reactor system developed in this study is expected to enable the utilization of CO_2_ and H_2_ obtained from other sources, such as industrial waste gases, biomass and gasified plastics. We plan to improve the stability of the enzymes for the long-term operation of the reactor and improve the cofactor co-immobilization system (Cha et al., 2023b) for implementation as a continuous reactor.

## Data Availability

The original contributions presented in the study are included in the article/Supplementary Material, further inquiries can be directed to the corresponding authors.
