# Supplementary material for: Real flue gas CO2 hydrogenation to formate by an enzymatic reactor using O2- and CO-tolerant hydrogenase and formate dehydrogenase
Source: Front Bioeng Biotechnol. 2023 Oct 3;11:1265272. doi: 10.3389/fbioe.2023.1265272 (PMC10579561; doi:10.3389/fbioe.2023.1265272)
Supplement: Supplementary file 1 [file DataSheet1.PDF]

Supplementary Information

Real Flue Gas CO_2 Hydrogenation to Formate by an Enzymatic Reactor Using O_2- and CO-Tolerant Hydrogenase and Formate Dehydrogenase

Jaehyun Cha[#1], Jinhee Lee[#2], Byoung Wook Jeon[#2], Yong Hwan Kim[2*], Inchan Kwon[1, 3*]

[1]School of Materials Science and Engineering, Gwangju Institute of Science and Technology (GIST), Gwangju, Republic of Korea

[2]School of Energy and Chemical Engineering, Ulsan National Institute of Science and Technology (UNIST), Ulsan, Republic of Korea.

[3]Research Center for Innovative Energy and Carbon Optimized Synthesis for Chemicals (Inn-ECOSysChem), Gwangju Institute of Science and Technology (GIST), Gwangju, Republic of Korea

[#]Contributed equally

[*]**Correspondence:**

Corresponding Author

metalkim@unist.ac.kr

inchan@gist.ac.kr

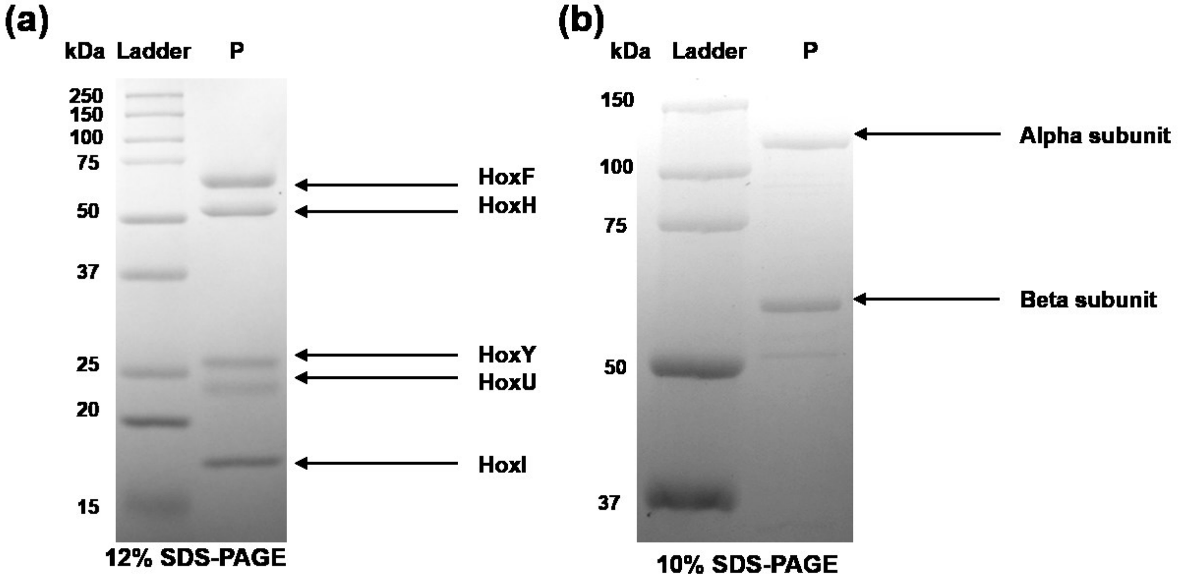

Figure S1. SDS-PAGE of purified proteins. (A) ReSH and (B) MeFDH1 stained with Coomassie blue. The lanes are molecular weight markers (Ladder), and purified protein (P).

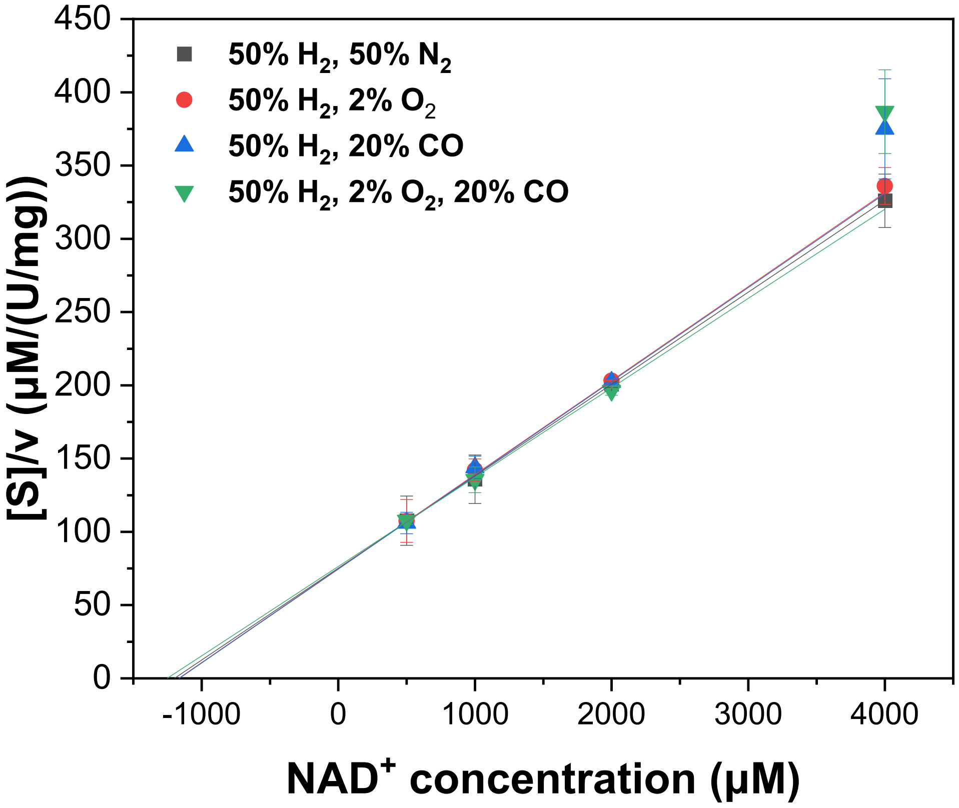

Figure S2. Hanes-Woolf plot for calculating the kinetic parameters of NAD^+-dependent H_2 oxidation of ReSH in the presence of O_2 and CO or not.

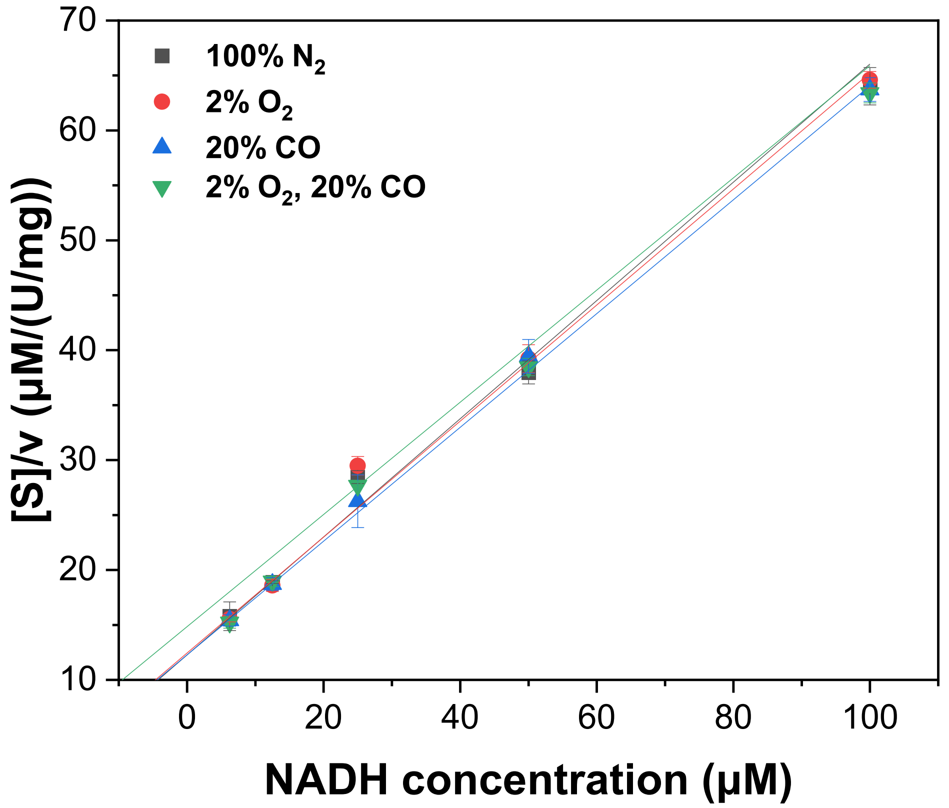

Figure S3. Hanes-Woolf plot for calculating the kinetic parameters of NADH-dependent CO_2 reduction of MeFDH1 in the presence of O_2 and CO or not.
